# Einfluss der Bildschirmzeit auf die Schlafqualität Studierender

**DOI:** 10.1007/s11818-022-00357-5

**Published:** 2022-06-01

**Authors:** Sophie Nestler, Irina Böckelmann

**Affiliations:** Bereich Arbeitsmedizin, Medizinische Fakultät, Otto-von-Guericke-Universität Magdeburg, Leipziger Str. 44, 39120 Magdeburg, Deutschland

**Keywords:** Schlafprävention, Mediennutzung, Chronotyp, Online-Lehre, COVID-19 Pandemie, Sleep prevention, Media usage, Chronotype, Online teaching, COVID-19 pandemic

## Abstract

**Einleitung:**

Die regenerative Wirkung des Schlafes ist für die körperliche, kognitive, emotionale und behaviorale Tagesleistung im Studium von essenzieller Bedeutung. Neben dem Stress im Studierendenalltag nimmt auch die Nutzung der Bildschirmgeräte im Vordergrund der pandemiebedingten Onlinelehre drastisch zu. Insbesondere in den Abend- und Nachtstunden kann die erhöhte Bildschirmnutzung zu einer physischen, psychischen und kognitiven Aktivierung beitragen, was wiederum die Schlafqualität der Studierenden negativ beeinflussen kann.

**Methoden:**

Zur Ermittlung möglicher Zusammenhänge zwischen der Bildschirmnutzung in der Onlinelehre sowie deren Auswirkungen auf die Schlafhygiene und -qualität nahmen 216 Studierende der Hochschule Magdeburg-Stendal anonym und freiwillig an der Onlinebefragung im Mai 2021 teil. Das positive Ethikvotum liegt vor.

**Ergebnisse:**

Die allgemeine Schlafqualität der Studierendenschaft ist bei 68,9 % (*n* = 149) der befragten Studierenden als schlecht zu bewerten. Besonders Studierende, die vorwiegend am Abend oder in der Nacht für ihr Studium an Bildschirmgeräten arbeiteten und somit chronobiologisch eine Tendenz zu einem Abendtyp aufwiesen, neigten zu einem schlechteren Schlaf.

**Diskussion:**

Die Auswirkungen langer Bildschirmzeiten in den Abend- und Nachtstunden können weitreichende Folgen für den Schlafrhythmus und das Bewusstsein für die körperlichen Bedürfnisse nach Pausen und Schlaf haben. Besonders die Onlinelehre in Zeiten der Coronapandemie führte zu einer Erhöhung der Bildschirmzeit neben der ebenso hohen privaten Nutzung. Daraus könnte eine weiterhin negative Beeinträchtigung der Schlafhygiene und -qualität nicht nur auf Kosten der Konzentrations- und Leistungsfähigkeit am Tage, sondern vielmehr der physischen und psychischen Gesundheit resultieren.

## Abstract (en)

**Introduction:**

The restorative effects of sleep are essential for physical, cognitive, emotional, and behavioral daily performance in college. In addition to the stress of everyday student life, the use of screen devices at the forefront of pandemic-related online teaching is also increasing dramatically. Particularly during evening and nighttime hours, increased screen use may contribute to physical, psychological, and cognitive activation, which in turn may negatively affect students’ sleep quality.

**Methods:**

To determine possible associations between screen use in online teaching and its effects on sleep hygiene and quality, 216 students at Magdeburg-Stendal University of Applied Sciences anonymously and voluntarily participated in the online survey in May 2021. The positive ethics vote is available.

**Results:**

The general sleep quality of the student body can be rated as poor in 68.9% (*n* = 149) of the surveyed students. In particular, students who worked predominantly on screen devices in the evening or at nighttime for their studies, and thus had a chronobiological tendency to be a night person, tended to have poorer sleep.

**Discussion:**

The effects of long screen time in the evening and nighttime hours can have far-reaching consequences on sleep patterns and awareness of physical needs for breaks and sleep. Online teaching in times of the COVID-19 pandemic in particular led to an increase in screen time alongside equally high levels of personal use. This could result in a continued negative impact on sleep hygiene and quality, not only at the expense of daytime concentration and performance but rather of physical and mental health.

## Einleitung

Ausreichender und erholsamer Schlaf trägt nicht unwesentlich zum Erhalt der psychischen und physischen Gesundheit bei [1]. Die Regenerationsprozesse beeinflussen dabei positiv die körperliche, kognitive, emotionale und behaviorale Tagesleistung [2] und sind somit für den Lernerfolg im Studium essenziell. Einen wesentlichen Einfluss auf die Schlafqualität dürfte eine Bildschirmnutzung im Alltag der Studierenden haben [3]. Das Sommersemester 2021 fand an der Hochschule Magdeburg-Stendal in Form von Lehre, Gruppenleistungen und Selbststudienanteilen ausschließlich online statt [4]. Besonders die Onlinelehre in Zeiten der Coronapandemie erhöht die Bildschirmzeit neben der ebenso hohen privaten Nutzung enorm, was wiederum die Schlafqualität der Studierenden negativ beeinflussen kann.

## Hintergrund und Fragestellung

Der aktuelle Forschungsstand an den einzelnen Hochschulen und Universitäten gab bereits ein klares Bild über Schlafverhalten und -qualität ihrer Studierendenschaft [3, 5]. Neben den Faktoren der Lebens‑, Sozial- und Wohnsituation gehören auch studienbezogene Faktoren, wie akademische Herausforderungen oder Prüfungs- und Leistungsdruck [6], zu den schlafbeeinträchtigenden Einflüssen des Studierendenlebens. Jedoch fehlen ausreichende Kenntnisse zu den Auswirkungen der Onlinelehre in der Zeit der Coronapandemie auf die Schlafqualität. Demnach könnten pandemiebedingt Aspekte der sozialen und digitalen Isolation, der finanziellen Unsicherheit durch fehlende Nebenerwerbstätigkeiten oder fehlende bzw. ungeeignete Studienarbeitsplätze eine nicht zu unterschätzende schlafbeeinträchtigende Rolle spielen.

**Figure 1:**
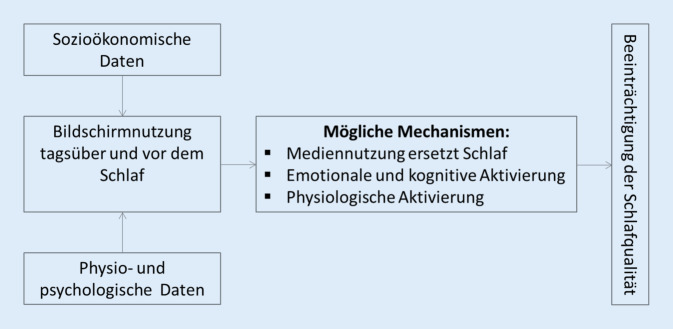

Demnach wird das nachfolgende Modell zur Mediennutzung und Schlaf zugrunde gelegt [7]. Nach Cain und Gradisar (2010) kann besonders die Bildschirmzeit (d. h. die Dauer der Nutzung der Bildschirmgeräte bei der Arbeit sowie die Tageszeit und die unterschiedlichen privaten und studienbezogenen Zwecke des Einsatzes der Bildschirmgeräte) ausschlaggebend sein. Die resultierende kognitive, psychische und physische Erregung („arousal“) kann vielmehr das Einschlafen erschweren und weiterhin einen negativen Einfluss auf die Schlafqualität ausüben. Unter Betrachtung der Mediennutzung nehmen Friedrich und Co-Autoren (2016) an, dass sich an Wochentagen mit festen Aufstehzeiten infolgedessen die Schlafdauer weiterhin verkürzt und langfristig zu einer ernst zu nehmenden Beeinträchtigung der Schlaf- und der damit verbundenen Lebensqualität führt ([5]; Abb. 1).

Da das Modell nach Cain und Gradisar (2010) sich auf die Mediennutzung Jugendlicher bezieht, gilt es dies an die Kontext- und Lebensbedingungen Studierender anzupassen.

Es wird sich demnach mit dieser quantitativen Querschnittstudie zum Ziel gesetzt, das Bildschirmnutzungsverhalten *im Sommersemester 2021 (ausschließlich Onlinelehre, bestehend aus virtuellen Pflichtveranstaltungen, Gruppenarbeiten und Selbststudium) *und dessen Auswirkungen auf die Schlafhygiene und -qualität der Studierenden einer Hochschule zu untersuchen.

Einerseits soll ein allgemeines Bild zu Schlafqualität und -verhalten der Studierenden verschafft werden. Andererseits wird erwartet, dass eine Reihe von Faktoren durch die Onlinelehre einen Einfluss auf die Schlafqualität der Studierenden hat. Zielsetzend sollen statistische Zusammenhänge zwischen den potenziellen Einflussgrößen, wie Bildschirmnutzung an Wochentagen/-enden, Neigung der individuellen zirkadianen Phasenlage (Chronotyp), auf Grundlage des Modells nach Cain und Gradisar (2010) auf die Schlafqualität festgestellt werden.

## Studiendesign und Untersuchungsmethoden

An der Untersuchung nahmen 319 Teil- und Vollzeitstudierende aller Fachbereiche, die zum Sommersemester 2021 an der Hochschule Magdeburg-Stendal immatrikuliert waren (135 weiblich, 43 männlich und 2 divers), im Alter von 18 bis 48 Jahren (mit einem Durchschnittsalter von 24,1 ± 5,14 Jahren) freiwillig und anonym teil. Aus dem Erhebungszeitraum im Mai 2021 gingen mittels des Online-Befragungstools SoSciSurvey 216 vollständig auswertbare Datensätze in die Auswertung ein.

Zur Darstellung der Bildschirmnutzung und der Schlafqualität wurde jeweils ein Onlinefragebogen mit einer geschätzten Bearbeitungszeit von 25 min eingesetzt. Die Zusammenstellung des Fragebogens orientierte sich an dem Modell von Cain und Gradisar (2010). Demnach wurden im ersten Teil des Fragebogens die subjektive Einschätzung des Umfangs und die Verteilung des Bildschirmnutzungsverhaltens erhoben. Die Bildschirmnutzung wurde differenziert an Werktagen als auch an Samstagen, Sonntagen und anderen freien Tagen erhoben. Es wurde ein Beobachtungszeitraum von vier Wochen analog zum PSQI-Fragebogen gewählt. Zur Beantwortung der Fragen zur Häufigkeit der Bildschirmnutzung wurde vorwiegend eine fünfstufige Antwortskala verwendet: „(fast) immer“ – „oft“ – „gelegentlich“ – „selten“ – „(fast) nie“.

Im zweiten Teil des Fragebogens wurde die Schlafqualität der Studierenden anhand des standardisierten Verfahrens Pittsburgh-Sleep-Quality-Index (PSQI) erfragt [8, 9]. Der PSQI erfasst retrospektiv in insgesamt sieben Komponenten, neben der subjektiven Einschätzung der Schlafqualität, die Schlaflatenz, -dauer und -effizienz sowie mögliche Schlafstörungen, den Schlafmittelkonsum und die Tagesschläfrigkeit. Diese sieben Komponenten fassen insgesamt 19 Items zur Selbstbeurteilung, welche jeweils einen Wertebereich von null bis drei annehmen können. Zusätzlich wurden fünf Fragen zur Fremdbeurteilung durch bspw. eine*n Partner*in hinzugefügt, welche allerdings nicht in die folgende Bewertung mit eingehen. Die Summe des Komponentenscores ergibt schließlich einen Gesamtscore, welcher im Wert von 0 bis 21 variieren kann. Der empirisch bestimmte Cut-off-Wert (> 5) erlaubt eine Einteilung in „gute“ und „schlechte“ Schläfer. Die Erfassung der subjektiven Schlafqualität bezieht sich nach Buysse et al. (1989) auf die letzten vier Wochen, wobei in der deutschen Fassung ein Zeitraum von lediglich zwei Wochen verwendet wird [10]. In dem in dieser Untersuchung verwendeten Fragebogen sind aus Zwecken der Vereinheitlichung des Zeitraums die letzten vier Wochen betrachtet worden.

Der dritte Teil des Fragebogens diente der Feststellung physio- und psychologischer Faktoren nach dem Horne-Östberg-Fragebogen zum Chronotyp (D-MEQ) [11]. Dazu wurde der von Horne und Östberg (1976) entwickelte Morningness-Eveningness Questionnaire für den deutschen Sprachraum aufbereitet und validiert. Da der Studierendenalltag im Onlinesemester durch flexible Arbeitszeiten in Selbststudium, Lehre oder Gruppenarbeiten geprägt ist, soll geprüft werden, ob die Studierenden die Arbeitszeit entsprechend ihrer zirkadianen Phasenlage (Chronotyp) verteilten. Es wird zwischen den folgenden fünf Ausprägungen unterschieden: definitiver Morgentyp, moderater Morgentyp, Normaltyp, moderater Abendtyp und definitiver Abendtyp [11]. Für die vorliegende Version ist besonders die Unterteilung in den Morgen- und den Abendtyp von Relevanz.

Ein Auswertungsbogen ordnet den verschiedenen Antwortmöglichkeiten entsprechende Bewertungsziffern zu. Auf die Addition der einzelnen Ziffern mit einem maximalen Summenscore von 86 folgt nach Griefahn et al. (2001) folgende fünftstufige Kategorisierung:

- definitiver Abendtyp (14–30)
- moderater Abendtyp (31–41)
- Neutraltyp (42–58)
- moderater Morgentyp (59–69)
- definitiver Morgentyp (70–86)

Abschließend werden im letzten Teil des Fragebogens personenbezogene Faktoren abgefragt. Demnach werden neben Alter und Geschlecht in erster Linie medizinische Daten, wie Körpergröße und Gewicht zur Berechnung des Body-Mass-Index (BMI) erfasst. Neben Angaben zum Studium (Studienform, -abschluss sowie Fachbereich und -semester) werden auch Aspekte zur Nebenbeschäftigung und Wohnsituation berücksichtigt. Auch Angaben zu einem potenziellen Partner oder der allgemeinen familiären Situation mit Kindern können einen erheblichen Einfluss auf das Schlafverhalten der Probanden haben und dürfen daher nicht unberücksichtigt bleiben. Das positive Votum der Ethikkommission der Medizinischen Fakultät an der Otto-von-Guericke-Universität liegt vor (Reg.-Nr. 54/21).

## Statistik

Die Aufbereitung der Daten und die statistische Auswertung erfolgten mit dem Statistik- und Analyseprogramm IBM SPSS Statistics 26, IBM, Armonk, NY, USA.

In den ersten Schritten der Auswertung wurden die Mittelwerte (MW) und Standardabweichungen (SD) sowie Mediane und Spannweiten (bzw. Range) im Rahmen der deskriptiven Statistik ermittelt. Für die Auswertung wurde ein Signifikanzniveau von 5 % festgelegt, wobei Signifikanzniveaus folgendermaßen definiert sind:

* signifikanter Unterschied (*p* < 0,05),
** sehr signifikanter Unterschied (*p* < 0,01) und
*** hoch signifikanter Unterschied (*p* < 0,001).

Der Kolmogorov-Smirnov-Anpassungstest wurde zur Prüfung von Variablen auf Normalverteilung herangezogen. Nachfolgend ist als parametrischer Test der Zweistichproben-t-Test für unabhängige Stichproben für normalverteilte und intervallskalierte Variablen genutzt worden. Für ordinalskalierte oder nicht normalverteilte intervallskalierte Variablen hingegen wurde der Mann-Whitney-U-Test verwendet. Bei multiplen Mittelwertvergleichen wurde die Bonferroni-Korrektur durchgeführt, um der Alphafehler-Kumulierung entgegenzuwirken. Die signifikanten Ergebnisse wurden stets fett in den Ergebnistabellen hervorgehoben.

Um die Zusammenhänge zwischen den wesentlichen Variablen zur Bildschirmnutzung und dem Chronotyp zu ermitteln, wurde eine Korrelationsanalyse durchgeführt. Da diese Variablen nicht normalverteilt waren, wurde die Rangkorrelation nach Spearman genutzt. Die Interpretation der Stärke bemisst sich nach der folgenden Einteilung, wobei das Vorzeichen jeweils für einen positiven bzw. negativen Zusammenhang steht:

- sehr geringe Korrelation (r < 0,2),
- geringe Korrelation (r < 0,5),
- mittlere Korrelation (r < 0,7),
- hohe Korrelation (r < 0,9) und
- sehr hohe Korrelation (r > 0,9).

## Ergebnisse

Von insgesamt 319 teilgenommenen Probanden wurden 216 auswertbare Datensätze durch einen vollständigen PSQI-Fragebogen (Durchschnittsalter von 23,9 ± 5,17 Jahre; von 18 bis 57 Jahre) generiert, wobei es durch nicht vollständig ausgefüllte Fragebögen zu Variationen in der Anzahl der Probanden in den Untergruppen kommen kann. Die 161 weiblichen Teilnehmenden waren im Durchschnitt 23,9 ± 5,49 Jahre (18–57 Jahre), die 51 männlichen Teilnehmenden 24,3 ± 4,16 Jahre (18–38 Jahre) und die zwei diversen Teilnehmenden 28 ± 7,07 Jahre (23 und 33 Jahre) alt. Zwei Teilnehmende sind ohne Angaben zum Geschlecht zu verzeichnen.

**Figure 2:**
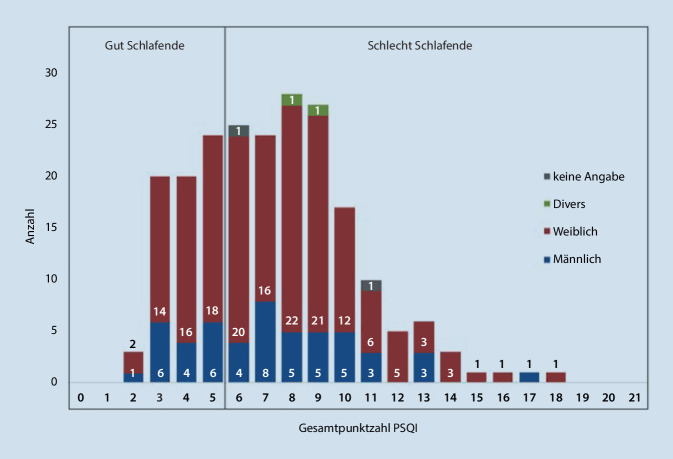

Der Gesamtscore des PSQI-Fragebogens diente der Gruppeneinteilung: der Cut-off-Wert (> 5) erlaubt eine Einteilung in „gute“ und „schlechte“ Schläfer nach Buysse et al. (1989). Die Abb. 2 zeigt die absolute Häufigkeit der im PSQI erreichten Gesamtpunktzahlen der befragten Studierenden.

Die Gesamtpunktzahl erreichte in der untersuchten Stichprobe Werte von 2 bis 18 Punkten, wobei eine Punktzahl von 0 bis 21 erreicht werden konnte. Insgesamt wiesen 31 % der Studierenden eine gute Schlafqualität auf. Bei 69 % der Studierenden wurde die Schlafqualität als schlecht bewertet.

In der Tab. 1 sind die einzelnen Komponenten des PSQI (subjektive Schlafqualität, Schlaflatenz, Schlafdauer, Schlafeffizienz, Schlafstörungen, Schlafmittelkonsum und Tagesschläfrigkeit) mit jeweils einem Wert von 0 bis 3 nach guter und schlechter Schlafqualitätsgruppe aufgeschlüsselt dargestellt. Die Komponente Schlafstörungen beinhaltet nächtliche Beschwerden, wie Einschlafprobleme, nächtliches Aufwachen, Harndrang, Atemprobleme, Husten oder Schnarchen, Kälte oder Wärme, Albträume oder Schmerzen.

**Table 1:** 

	Gut Schlafende (*n* = 67)	Schlecht Schlafende (*n* = 149)	Gesamt (*n* = 216)	*p*-Wert
	MW ± SD Median (Range)	MW ± SD Median (Range)	MW ± SD Median (Range)	
Gesamtpunktzahl PSQI	4,0 ± 0,92 4 (2–5)	8,8 ± 2,39 8 (6–18)	7,3 ± 3,04 7 (2–18)	**<** **0,001**
Subjektive Schlafqualität	0,9 ± 0,44 1 (0–2)	1,7 ± 0,58 2 (1–3)	1,4 ± 0,65 1 (0–3)	**<** **0,001**
Schlaflatenz	0,7 ± 0,61 1 (0–2)	2,0 ± 0,88 2 (0–3)	1,6 ± 1,01 2 (0–3)	**<** **0,001**
Schlafdauer	0,2 ± 0,41 0 (0–1)	0,8 ± 0,66 1 (0–3)	0,6 ± 0,65 1 (0–3)	**<** **0,001**
Schlafeffizienz	0,1 ± 0,24 0 (0–1)	0,8 ± 0,87 1 (0–3)	0,6 ± 0,81 0 (0–3)	**<** **0,001**
Schlafstörungen	1,0 ± 0,24 1 (0–2)	1,5 ± 0,54 1 (1–2)	1,4 ± 0,52 1 (0–2)	**<** **0,001**
Schlafmittelkonsum	0,0 ± 0,00 1 (1–2)	0,2 ± 0,64 1 (0–3)	1,3 ± 0,54 1 (0–3)	**0,007**
Tagesschläfrigkeit	1,1 ± 0,55 1 (0–2)	1,9 ± 0,72 2 (0–3)	1,7 ± 0,76 2 (0–3)	**<** **0,001**

*p*-Wert: Mann-Whitney-U-Test

Signifikanz: *p* < 0,05 (fett dargestellt)

Durch die Verwendung der erzielten Gesamtpunktzahl für die Gruppeneinteilung kommt dem abgebildeten *p*-Wert keine hohe Bedeutung zu.

## Einfluss soziodemografischer Faktoren

Eine gute Schlafqualität erwies sich bei 67 Studierenden, davon 74,6 % weiblich und 25,4 % männlich. Bei 147 Befragten (75,5 % weiblich, 23,1 % männlich und 1,4 % divers) hingegen wurde die Schlafqualität als schlecht bewertet. Es besteht eine vergleichbare Geschlechtsverteilung in den Schlafqualitätsgruppen (p_x_^2^ = 0,602).

In Hinblick auf das Alter, den BMI und die studienbezogene Auslastung wurde zunächst auf Unterschiede bei den Schlafqualitätsgruppen (gut und schlecht Schlafende) geprüft (Tab. 2).

**Table 2:** 

	Gut Schlafende (*n* = 67)	Schlecht Schlafende (*n* = 148)	Gesamt (*n* = 215)	*p*-Wert
	MW ± SD Median (Range)	MW ± SD Median (Range)	MW ± SD Median (Range)	
Alter [Jahre]	24,1 ± 5,14 23 (18–48)	24,0 ± 5,25 23 (18–57)	24,0 ± 5,2 23 (18–57)	0,793
BMI [kg/m^2^]	23,1 ± 3,78 22,3 (18–34)	23,9 ± 4,72 22,9 (15–48)	23,7 ± 4,46 22,8 (15–48)	0,246
Nebentätigkeit [Stunden/Woche]	14,9 ± 12,14 10 (0–40)	13,6 ± 9,48 10 (2–42)	14,0 ± 10,43 10 (0–42)	0,917
Studienaufwand wochentags [Stunden/Tag]	6,0 ± 3,38 6 (1–20)	6,3 ± 2,65 6 (0–15)	6,2 ± 2,89 6 (0–20)	0,437
Studienaufwand Wochenende [Stunden/Tag]	3,1 ± 2,35 3 (0–10)	4,2 ± 3,11 4 (0–20)	3,8 ± 2,93 3 (0–20)	**0,031**

*p*-Wert: Mann-Whitney-U-Test

Signifikanz: *p* < 0,05 (fett dargestellt)

Die Studierenden sind nach der BMI-Klassifizierung der WHO (2000) im Durchschnitt als normalgewichtig (entsprechend 18,50 kg/m^2^ bis 24,99 kg/m^2^) einzustufen. Des Weiteren waren in der Gruppe der gut schlafenden Studierenden 4,5 % mit Kindern vertreten, während der Anteil der studierenden Eltern in der Gruppe der schlechteren Schlafqualität 4,1 % betrug. Ein signifikanter Unterschied zwischen den beiden Gruppen konnte jedoch nicht festgestellt werden (p_x_^2^ = 0,894).

## Einfluss des Chronotyps

Nach dem Horne-Östberg-Fragebogen zum Chronotyp (D-MEQ) (Griefahn et al., 2001) wurden 5 Chronotypen kategorisiert. In Betrachtung der erreichten Gesamtpunktzahl der befragten Studierenden ergibt sich im Durchschnitt der Wert 50,7, was dem Neutraltyp (mit 42–58 Punkten) entspricht.

**Figure 3:**
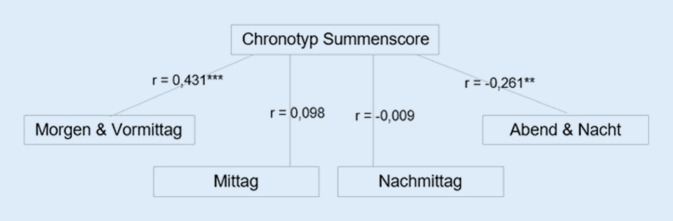

In Betrachtung der Präferenzen zum Studienaufwand zu verschiedenen Tageszeiten konnten signifikante Zusammenhänge zwischen der Gesamtpunktzahl des Fragebogens zum Chronotyp und den frühen und späten Tageszeiten der Bildschirmnutzung für die Studienzwecke festgestellt werden (Abb. 3).

Das bestätigt den Zusammenhang, dass Studierende mit der Tendenz zum Morgentyp sich auch eher am Morgen und Vormittag dem Studium widmen. Studierende, die eher am Abend und in der Nacht für ihr Studium arbeiten, haben hingegen eine Tendenz zum Abendtyp.

**Figure 4:**
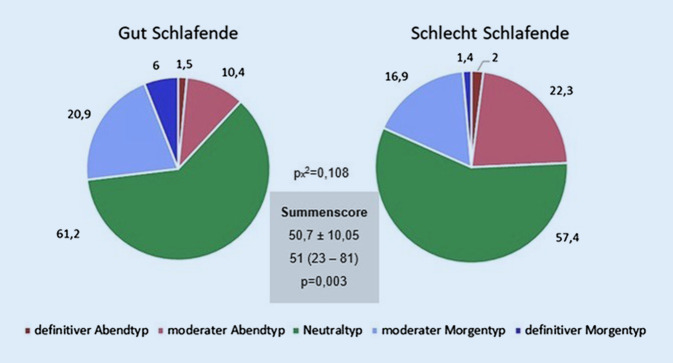

Hinsichtlich der Schlafqualität ergibt sich ein signifikanter Gruppenunterschied, wobei gut schlafende Studierende mit einer höheren Gesamtpunktzahl im Vergleich zum Gesamtdurchschnitt zum Morgentyp tendieren (Abb. 4). Die Studierenden mit einer schlechteren Schlafqualität hingegen weisen eine niedrigere Punktzahl auf und tendieren somit eher zum Abendtyp:

## Einfluss der Bildschirmnutzung

An einem gewöhnlichen Wochentag im Semester 2021 der letzten vier Wochen arbeiteten die Studierenden im Durchschnitt 6,2 h. Dabei konnte kein signifikanter Unterschied zwischen den Gruppen festgestellt werden, jedoch arbeiteten die Studierenden mit einer schlechten Schlafqualität tendenziell mehr. In Anbetracht der Verteilung des zeitlichen Studienaufwandes über den Tag konnten signifikante Unterschiede bei den Abendstunden ab 17 bis 06 Uhr festgestellt werden. Schlussfolgernd litten Studierende, welche besonders am Abend und in der Nacht Studienarbeiten erledigten, unter einer schlechteren Schlafqualität.

An studienarbeitsfreien Tagen des Sommersemesters 2021 arbeiteten die Studierenden im Durchschnitt 3,8 h. Dabei konnte ein signifikanter Unterschied zwischen den Gruppen festgestellt werden, wobei die Studierenden mit einer schlechten Schlafqualität im Durchschnitt etwa eine Stunde mehr für ihr Studium arbeiteten als gut schlafende Studierende. In Anbetracht der Verteilung des zeitlichen Studienaufwands über den Tag konnten signifikante Unterschiede bei den Abendstunden ab 14 bis 06 Uhr (*p* = 0,001) an Wochentagen festgestellt werden. Schlussfolgernd litten Studierende, welche besonders am Abend und in der Nacht Studienarbeiten erledigten, unter einer schlechteren Schlafqualität. Im Vergleich zur Verteilung des zeitlichen Studienaufwandes an Samstagen, Sonntagen oder anderen freien Tagen sind Gruppenunterschiede bereits bei studienbezogenen Aufwänden ab dem Nachmittag (*p* = 0,005) und besonders in den Abend- (*p* ≤ 0,001) und Nachtstunden (*p* = 0,001) zu verzeichnen.

Des Weiteren wurden die Studierenden befragt, wie häufig Bildschirmgeräte genutzt wurden, wenn sie nachts aufgewacht sind. Dabei sind höchst signifikante Unterschiede in den Schlafqualitätsgruppen zu verzeichnen. Knapp die Hälfte (49,1 %) der schlecht schlafenden Studierenden nutzten zumindest gelegentlich, oft oder immer Bildschirmgeräte, wenn sie nachts aufwachten (*p* ≤ 0,001). Bei der Gruppe der gut schlafenden Studierenden schauten nachts hingegen 26,9 % auf die Bildschirmgeräte. Über eine Filterfunktion wurden diese Studierenden zu einer Frage zu den Zwecken der nächtlichen Bildschirmgerätenutzung weitergeleitet.

Dabei unterschieden sich die Schlafqualitätsgruppen in studienbezogenen Zwecken (für das Studium etwas nachschauen, fertigstellen oder notieren), wobei schlecht schlafende Studierende häufiger zu diesen Zwecken Bildschirme in der Nacht nutzten als Studierende mit einer guten Schlafqualität.

Neben dem negativen Einfluss auf die Schlafqualität zeigt sich, dass die Nutzung eines Smartphones mit unscharfem Sehen (p_M‑W_ = 0,009) und Kopfschmerzen (p_M‑W_ = 0,006) in Verbindung gebracht werden konnte. Ein weiterer Zusammenhang besteht zwischen der Nutzung des Laptopmonitors und Verspannungen im Schulter-Nacken-Bereich (p_M‑W_ = 0,002). Beanspruchungen der Augen (d. h. unscharfes Sehen p_x_^2^ = 0,004, Augenbrennen und -ermüdung p_x_^2^ = < 0,001) sowie des Kopf-Nacken-Bereiches, in Form von Kopfschmerzen (p_x_^2^ = < 0,001) und Verspannungen (p_x_^2^ = 0,010), treten signifikant häufiger bei Studierenden mit schlechter Schlafqualität auf.

## Diskussion

Ausreichender und erholsamer Schlaf ist nicht nur ein wesentlicher Bestandteil der psychischen und physischen Gesundheit [12], sondern trägt erheblich zur allgemeinen Leistungsfähigkeit bei. In einer Befragung von 216 Studierenden konnte bei 59 % eine Beeinträchtigung der Schlafqualität festgestellt werden [3]. Ähnliche Ergebnisse zeigt auch diese Studie: Bei 69 % der Teilnehmenden wurde die Schlafqualität als schlecht bewertet. Während in einer Befragung von 307 Studierenden der Anteil der schlecht Schlafenden vor der Covid-19-Pandemie bei 58 % lag, erhöhte sich dieser Anteil um 15,3 % während der Covid-19-Pandemie [13].

Eine Verschlechterung der Schlafqualität kann mit einem Defizit in der Konzentrationsfähigkeit, Stress und schlechterem psychischen Wohlbefinden in Verbindung gebracht werden [14–18]. Die Studienergebnisse sprechen dafür: Die Studierenden mit einer schlechten Schlafqualität arbeiteten im Durchschnitt etwa eine Stunde mehr für ihr Studium als gut schlafende Studierende.

Als Gründe dafür könnten die studienbezogenen Belastungsfaktoren sowie Faktoren aus der Lebens‑, Sozial- und Wohnsituation benannt werden. Alle hier diskutierten Studien wurden in der präpandemischen Zeit durchgeführt. Seit der Pandemie sind zusätzliche potenziell schlafbeeinträchtigende Faktoren im Studierendenleben, wie die Erhöhung der Bildschirmnutzung oder digitale und soziale Isolation, hinzugekommen. In der hier durchgeführten Studie werden nicht alle diese Faktoren, zum Beispiel die Wirkung der sozialen und digitalen Isolation, die Beschaffung der Studienarbeitsplätze oder finanzielle Risiken, erhoben worden, was als Limitation zu sehen ist. Ob nun die Schlafbeeinträchtigungen ausschließlich auf die Bildschirmnutzung in dem Onlinesemester oder auf die pandemischen Faktoren zurückzuführen sind, kann anhand dieser Studie nicht beantwortet werden.

Die Ergebnisse der hier vorgestellten Studie verdeutlichen, dass Studierende mit der Tendenz zum Morgentyp sich auch in der ersten Hälfte des Tages dem Studium widmen. Studierende, die sich eher in späteren Stunden (Abend und Nacht) mit den studienbezogenen Aufgaben beschäftigten, haben hingegen eine Tendenz zum Abendtyp.

Insgesamt ist während der Covid-19-Pandemie in der Gesamtbevölkerung eine Verschiebung der Tag-Nacht-Rhythmik zu erkennen. Generell konnte in Studien belegt werden, dass die Homeofficebedingungen, z. B. durch den Wegfall der Arbeitswege, die Schlafdauer verlängerten [19, 20] Zusätzlich passten sich die Arbeits- und Wachzeiten besser an den biologischen Rhythmus an [21].

Auf der anderen Seite ist ein deutlicher Anstieg der Bildschirm- bzw. Mediennutzung zu erkennen: Während sich die durchschnittliche tägliche Nutzungsdauer bei den 18- bis 29-Jährigen im Zeitraum 2012–2013 noch auf 3:04 h belief, stieg die Nutzungszeit in der Pandemie auf 7:15 h [22].

Lange Bildschirmzeiten, besonders in den Abendstunden, können den natürlichen Schlafrhythmus verschieben und sorgen dafür, dass das Bewusstsein für die körperlichen Bedürfnisse nach Pausen und Schlaf abnimmt [23]. Besonders die Onlinelehre in Zeiten der Coronapandemie sorgte enorm für die Erhöhung der Bildschirmzeit neben der ebenso hohen privaten Nutzung, um dem Social Distancing entgegenzuwirken. Dies wiederum beeinflusst die Schlafqualität der Studierenden weiterhin negativ und erhöht schließlich die Tagesschläfrigkeit auf Kosten der Konzentrationsfähigkeit [24]. Besonders die nächtliche Bildschirmnutzung bei fast der Hälfte der schlecht schlafenden Studierenden (49,1 %) zeigt die Notwendigkeit einer Sensibilisierung zur Mediennutzung und für Strategien zur Schlafhygiene.

Als mögliche Strategie zur Bildschirmlichtreduzierung wurden die Studierenden bezüglich der Nutzung eines Nachtlichtmodus und eines Blaulichtfilters befragt. Lediglich jede*r dritte*r Studierende nutzte den Nachtlichtmodus zur Reduzierung des Blaulichtanteils bei allen digitalen Geräten (30,9 %) oder eine Sehhilfe mit Blaulichtfilter (31 %). Aktuelle Studien zeigen jedoch, dass die Wirkung von LED-Displays auf den Schlaf nicht bestätigt werden konnte [25].

Dennoch stellt eine erhöhte Bildschirmnutzung ein potenzielles Risiko für die physische Gesundheit dar: Einerseits traten Schulter-Nacken-Beschwerden [26–28] und andererseits Ermüdungserscheinungen der Augen [29, 30] auf. Das Auftreten gleicher Beschwerden wurde in dieser Befragung bestätigt.

In diesem Sinne spielt die Gesundheitsförderung und Prävention an der Hochschule mit deren hoher Reichweite eine besondere Rolle, durch ihre Studierendenschaft als MultiplikatorInnen möglichst viele gesellschaftliche Arbeits- und Lebenskontexte anzustoßen und voranzubringen [5, 31].

## Fazit für die Praxis

- Die durchgeführte Studie bietet eine hohe Übertragungsmöglichkeit auf andere Kontexte in Bezug auf eine erhöhte, besonders mobile Bildschirmarbeitszeit. Insbesondere die Flexibilität des Einsatzes der Bildschirmgeräte gefährdet die Einhaltung einer notwendigen Schlafhygiene.
- Resultierend sind fachspezifische Empfehlungen für Studierende und Lehrende zu einer Reduzierung der Bildschirmzeit und zur weiteren Verbesserung der Schlafqualität und -hygiene zu formulieren. Darüber hinaus ist es unumgänglich, studiengangspezifische schlafbeeinträchtigende Belastungsfaktoren zu identifizieren und in Schlafpräventionsprogrammen zu implementieren. Weiterführende Ergebnisse können auch Aufschluss über zukünftige gesundheitsförderliche Konzepte für die Hochschulorganisation (hinsichtlich der Seminar- und Prüfungsplanung) geben, da akademische Herausforderungen durch den Leistungs- und Prüfungsdruck zu den beeinträchtigenden Faktoren in Bezug auf den Schlaf zählen. Jedoch besteht der Bedarf, die Abhängigkeit der Bildschirmzeit und somit der Schlafqualität auch im Verlauf der sich ändernden Studienbedingungen während des Semesters zu prüfen. Weiterhin ist es sinnvoll, Programme der Stressbewältigung mit schlafpräventiven Elementen zu ergänzen. Denn ab dem 01.07.2021 wurde das Thema „Schlaf“ zunächst für digitale Präventions- und Gesundheitsförderungsprogramme im Rahmen des Stressmanagements aufgenommen.
- Diese Programme werden gemäß dem Leitfaden Prävention von den Krankenkassen unterstützt. Darüber hinaus besteht ab dem 01.04.2022 sogar die Zertifizierungsmöglichkeit der „Förderung des gesunden Schlafes“. Die Befragung zeigt die erhöhte Nachfrage, vor allem der schlecht schlafenden Studierenden, nach Entspannungstechniken. Hierzu zählen beispielsweise Angebote der Stressbewältigung, der psychosozialen Beratung oder ähnliche Angebote der Hochschule Magdeburg, welche zukünftig modifiziert und präsenter gestaltet werden sollten.
